# New Insights Into Targeting Membrane Lipids for Cancer Therapy

**DOI:** 10.3389/fcell.2020.571237

**Published:** 2020-09-02

**Authors:** Giulio Preta

**Affiliations:** Institute of Biochemistry, Life Sciences Center, Vilnius University, Vilnius, Lithuania

**Keywords:** lipid rafts, membrane fluidity, cell signaling, amphiphilic molecules, protein receptors

## Abstract

Modulation of membrane lipid composition and organization is currently developing as an effective therapeutic strategy against a wide range of diseases, including cancer. This field, known as membrane-lipid therapy, has risen from new discoveries on the complex organization of lipids and between lipids and proteins in the plasma membranes. Membrane microdomains present in the membrane of all eukaryotic cells, known as lipid rafts, have been recognized as an important concentrating platform for protein receptors involved in the regulation of intracellular signaling, apoptosis, redox balance and immune response. The difference in lipid composition between the cellular membranes of healthy cells and tumor cells allows for the development of novel therapies based on targeting membrane lipids in cancer cells to increase sensitivity to chemotherapeutic agents and consequently defeat multidrug resistance. In the current manuscript strategies based on influencing cholesterol/sphingolipids content will be presented together with innovative ones, more focused in changing biophysical properties of the membrane bilayer without affecting the composition of its constituents.

## Introduction

Lipid-driven membrane organization is essential for the physiological functions of eukaryotic cells since it regulates a multitude of processes including intracellular signaling, redox balance and cell death (Muro et al., 2014; Santos and Preta, 2018). Behind these regulatory properties, there is the lipids capacity to laterally aggregate, forming highly dynamic and heterogeneous regions, referred to as lipid rafts. Lipid rafts are nanoscale membrane microdomains (<200 nm), particularly enriched in cholesterol and sphingolipids, that selectively recruit certain protein receptors (Simons and Toomre, 2000; Sezgin et al., 2017). Lipid rafts form microscopic domains (>300 nm) upon clustering induced by protein-protein or protein-lipid interaction. The raft model was supported by observation on artificial membrane models, demonstrating that certain lipids specifically tend to interact with others to generate large scale lateral domains (Simons and Vaz, 2004; Kaiser et al., 2009). The presence of plasma membrane specific organization has been observed across different organisms, ranging from bacteria to yeasts, providing further support for their biological significance (Kaiser et al., 2009; Henderson and Block, 2014; Lopez, 2015). Changes in the composition and organization of lipids have several effects on cellular functions, influencing signal transduction, membrane plasticity, and membrane trafficking. Plasma membrane cholesterol is one of the most important regulators of lipid organization, representing the majority (up to 90%) of the total cellular cholesterol and its levels in the cells are tightly regulated (de Duve, 1971; Lange et al., 1989). According to a recent study there are three pools of cholesterol in the plasma membrane: a labile pool, depleted by cholesterol-targeting agents, a sphingomyelin-bound pool and an essential pool, necessary for cell viability (Das et al., 2014). Only the cholesterol not sequestered by proteins or lipids can be transported in the endoplasmic reticulum (ER) where it binds to specific sensors, shutting down cholesterol synthesis and uptake (Infante and Radhakrishnan, 2017). The pathway between cholesterol removal from plasma membrane and its subsequent transport to the ER represents a field of extensive investigation aimed to identify specific transporters involved in the regulation of cholesterol homeostasis. Recent studies identified Aster/GRAMD1 as essential transporters of cholesterol into ER and regulating the cellular uptake of HDL-derived cholesterol (Sandhu et al., 2018; Naito et al., 2019). ORP2 protein was also identified as a unique transporter of cholesterol from ER to the plasma membrane (Wang et al., 2019). There is no doubt that this recent progress in understanding cholesterol homeostasis and metabolism set the basis for the development of current therapies based on cholesterol and lipids targeting. A decrease in membrane cholesterol has been observed to have beneficial effects against different pathological condition including cancer and neurodegenerative diseases (Simons et al., 1998; Canevari and Clark, 2007; Guardia-Laguarta et al., 2009; Barros et al., 2018; Chen et al., 2018; Gu et al., 2019). Cholesterol-targeting can be achieved via cholesterol depletion, sequestration or inhibition of synthesis. The first effect is observed using cyclodextrins, a group of chemical compounds extracting cholesterol from the plasma membrane and widely used in the biomedical field in different experimental settings (Zidovetzki and Levitan, 2007; Lopez et al., 2013; Mahammad and Parmryd, 2015). Cholesterol sequestration is the mechanism used by different pore forming agents, by the antibiotic filipin, amphotericin, and nystatin (Bittman and Fischkoff, 1972; Silva et al., 2006; Kaminski, 2014). Cholesterol sequestration also effectively reduces the ability of cholesterol to interact with other membrane constituents. Statins, a widely used class of lipid-lowering medications are the best representatives of the inhibitors of cholesterol synthesis (Stancu and Sima, 2001; Kuipers and van den Elsen, 2007). These include other compounds like bisphosphonates or zaragozic acid acting at different levels of the mevalonate pathway (Amin et al., 1992; Griffin et al., 2017).

It is relevant to underline that few chemical compounds can affect the lipid membranes by different mechanisms. The dynamin inhibitor Dynasore has been shown to influence both cholesterol transport on the cell membrane and cholesterol concentration (Girard et al., 2011; Preta et al., 2015a,b). Beyond the cholesterol-lowering effects of statins, cholesterol-independent or pleiotropic effects are reported, including the capacity to modify plasma-membrane organization and structure (Wang et al., 2008; Penkauskas et al., 2020). Studies using artificial model membranes showed that statins alter the nanomechanical stability of the bilayers, intercalating the lipid-water interface and increasing membrane heterogeneity (Redondo-Morata et al., 2016; Galiullina et al., 2019). A better understanding of how therapeutic agents affect the membrane organization and composition, led in the last years to the development of a new field, named membrane-lipid therapy (MLT). MLT involves the identification and optimization of drugs capable to modify membrane lipid structures for pharmaceutical applications (Escriba, 2006; Escriba et al., 2015). Due to the essential role of the plasma membrane in many physiological processes, it is expected that MLT will provide new treatments for a wide range of diseases, including oncological disorders, neurodegenerative diseases, diabetes and stroke (Escriba, 2017).

## MLT for Cancer Therapy: A Brief Outline

**FIGURE 1:**
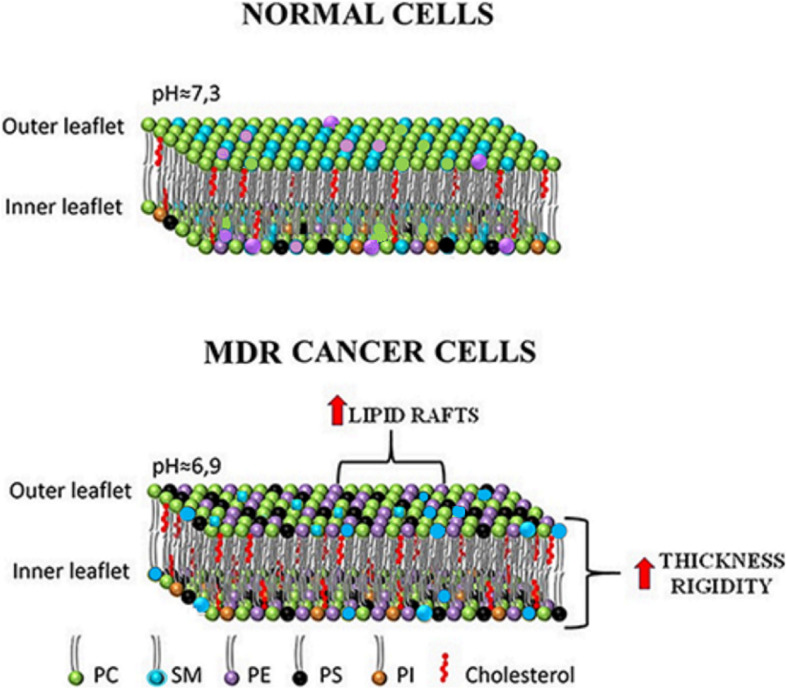
Differences between membrane lipid composition and organization in normal vs MDR cancer cells. In cancer cells PS and PE, mainly confined in the inner leaflet of the membranes, are present in high concentrations in the outer leaflet. Cancer cells have also higher concentrations of cholesterol and consequently an increase in membrane thickness and rigidity is observed. Increased levels of saturated fatty acyl chains in membrane lipids have been associated to the presence of more lipid rafts while low amount of ceramide in MDR cells is a consequence of the low activity of SMase or of the increased SM levels. Changes in lipid composition of the outer membrane of cancer cells are also correlated to a more acidic extracellular pH.

One of the hallmarks of cancer is the resistance to apoptosis and, more in general, the higher rate of proliferation versus death (Hanahan and Weinberg, 2000, 2011). The dynamicity of cell membranes plays an essential role in the regulation of cell surviving, through all the phases of a cell: lipid flexibility contributes to an increase in the mechanical stability during division and to a decrease of shear force during cell separation (Patra, 2008). To adapt rapidly, cancer cells re-organize their plasma membranes to preserve proliferation, escape apoptosis and resist to anticancer drugs treatment (Bernardes and Fialho, 2018). The latter is a crucial problem in anticancer therapy and often leads to multidrug resistance (MDR). Among many others, one of the causes of MDR is the decreased free diffusion of anticancer drugs through the plasma membrane. Therefore, the study and development of anticancer drugs capable to exert therapeutic effect by modulating the properties of tumor membranes, is constantly increasing (Rios-Marco et al., 2017; Zalba and Ten Hagen, 2017; Kopecka et al., 2020). The rationale for MLT is that there are fundamental differences in composition between normal and MDR cancer cells (Figure 1). MDR cells possess higher levels of total cholesterol as a result of an increased activity of HMG-CoA reductase, the rate-limiting enzyme in cholesterol synthesis (Kawata et al., 1990; Harwood et al., 1991). Additional studies reported an increase in mevalonate levels and in the expression of the low-density lipoprotein receptor compared to normal cells (Duncan et al., 2004; Kopecka et al., 2011). The observed higher amount of membrane cholesterol is responsible of a more rigid and less permeable membrane (Peetla et al., 2013; Niero et al., 2014). Moreover, MDR cells keep low ceramide levels by increasing sphingomyelin (SM) synthesis: this is an important anti-apoptotic strategy since implies a decrease in ceramide-enriched lipid rafts involved in the induction of cell death. Furthermore, phosphatidylserine (PS) and phosphatidylethanolamine (PE) which, under physiological conditions exist mainly in the inner leaflet of cell membranes have increased surface expressions on the outer membrane of tumor cells (Tan et al., 2017). The asymmetrical distribution of PS, maintained by a group of amino-phospholipid translocases that use ATP hydrolysis to flip PS from the external to the cytosolic leaflet, is also lost during the apoptotic process. The loss of PS asymmetry in cancer cells may be related to a reduced activity of these ATP-dependent phospholipid translocases or to an elevated activity of phospholipid scramblase, due to high levels of intracellular calcium (Ca^2+^_i_) (Chen et al., 1999). PS on the outer membrane of tumor cells can be used as an effective target for cancer therapy (Ran et al., 2002; Riedl et al., 2011; Davis et al., 2019). The PS-targeting antibody bavituximab (Chalasani et al., 2015; Gerber et al., 2015; Grilley-Olson et al., 2018) and the PS-binding peptide/peptoid hybrid PPS1D1 (Desai et al., 2016; Desai and Udugamasooriya, 2017) have shown significant cytotoxic effects in cancer cells. Another strategy largely used in anticancer therapy is to entrap the drug in a specific carrier, which held the tumor-targeting property (Dass and Choong, 2006; Fanciullino and Ciccolini, 2009). For example, a cationic liposomal carrier, phosphatidylcholine-stearylamine (PC-SA), strongly binds and kills cancer cells through direct interaction with negatively charged surface-exposed PS (De et al., 2018). The anticancer properties of drugs like camptothecin and doxorubicin entrapped in PC-SA liposomes was demonstrated on cancer cell lines, both *in vitro* and in different mice models (De et al., 2018, 2020). These and other studies showed the potential use in MLT of PS-targeting vesicle alone or in combination with anticancer drugs (Blanco et al., 2014; Ayesa et al., 2017). PE represents another chemotherapeutic target on the membrane surface of cancer cells. Duramycin is a small tetracyclic peptide produced by the bacterium *Streptoverticillium cinnamoneus* and is closely related to cinnamycin produced by *Streptomyces* sp. (Iwamoto et al., 2007; Hullin-Matsuda et al., 2016). Both duramycin and cinnamycin are capable to bind to PE specifically into areas of membrane with high curvature, inducing trans-bilayer phospholipid movements that lead to cell death (Makino et al., 2003; Iwamoto et al., 2007; Hullin-Matsuda et al., 2016). Another group of interesting molecules are cyclotides, cyclic peptides which exert their biological activities by acting on cell membrane, binding to phospholipids containing PE headgroups. This binding is followed by an insertion that subsequently leads to membrane disruption and cell death as a result of pore formation (Wang et al., 2012). The increased levels of exposed PE on the outer membrane of cancer cell allow those membrane-active peptides to exert their cytotoxic effects without harming healthy cells. A third target for MLT is ceramide. Ceramide is present in small amounts in cell membranes, as intermediate in the metabolism of sphingolipids or as a result of sphingomyelinase activity, which produces ceramide from SM (Kartal Yandim et al., 2013; Peetla et al., 2013). Altered ceramide metabolism in cancer has been described as an effective drug resistance mechanism: tumors have low levels of ceramide by increasing SM synthesis or by preventing its degradation (Senchenkov et al., 2001; Lewis et al., 2018). One possible strategy is to increase ceramide membrane levels using short chain ceramide and use lipid rafts as platforms to enhance apoptosis, since in presence of an excess of ceramide, cholesterol is displaced from lipid rafts, inducing activation of Fas/CD95 pathway (Selzner et al., 2001; Stover and Kester, 2003; Stover et al., 2005; Chiantia et al., 2007). Ceramide levels can also be increased by inhibiting the enzyme ceramidase (using the ceramide analogs B13, LCL-464 and KPB-27) or sphingosine kinase inhibitors (like N, N-dimethylsphingosine) (Bhabak and Arenz, 2012; Bhabak et al., 2013; Chen et al., 2014). Few reviews provide a complete list of compounds used in MLT based on regulation of ceramide levels (Lin et al., 2006; Kartal Yandim et al., 2013; Liu et al., 2013).

Activation of the Fas pathway is the target of treatment with different anticancer agents including Edelfosine, Miltefosine and Perifosine, lipid clustering agents promoting apoptosis (Gajate and Mollinedo, 2007; Gomide et al., 2013). Resveratrol, a common constituent of red wine, has been shown to have anti-tumor activity for its tendency to accumulate in lipid rafts and is mainly used in combination with death receptor agonists (Delmas et al., 2013). Azurin is a membrane-associated protein from *Pseudomonas aeruginosa*. Azurin and its derived peptide p28 have been intensively studied as an anticancer protein, down-regulating fundamental signaling pathways downstream of membrane receptors and affecting processes such as adhesion and invasiveness (Gao et al., 2017; Bernardes and Fialho, 2018). These effects are dependent on the caveolin 1 and ganglioside 1-mediated uptake of azurin, leading to alteration of lipid rafts; decrease in plasma membrane stiffness and in the number of ordered domains (Bernardes and Fialho, 2018). The increased sensitivity of cancer cells to chemotherapeutic agents like paclitaxel and doxorubicin in combination with azurin confirms that part of the anticancer effect of azurin occurs by altering the membrane properties and increasing the membrane permeability to anticancer drugs (Bernardes et al., 2018). However, the use of these peptides in MLT, has some limitations: they require further optimization to enhance their selectivity toward cancer cells and to decrease toxicity; in few cases their use alone or in combination with chemotherapeutic agents did not show any beneficial effect in clinical trials (Planting et al., 1993; Gills and Dennis, 2009; Cho et al., 2012; Gerber et al., 2018).

## Amphiphilic Molecules in Cancer Therapy

**FIGURE 2:**
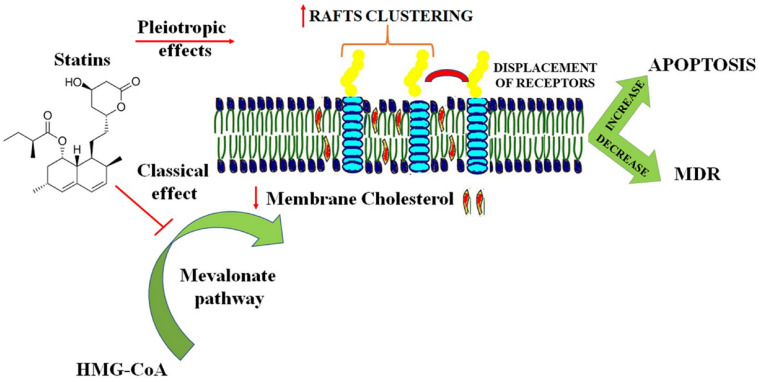
Different rationales behind the use of statins in cancer therapy for modulation of membrane lipids. The beneficial effects of statins in cancer therapy are achieved by a combination of classical and pleiotropic effects. The classical cholesterol lowering activity decreases the cholesterol concentration in the plasma membrane influencing membrane fluidity and thickness. Pleiotropic effects include lipid rafts clustering and displacement of receptors in non-raft domains. All these effects contribute to an increase in apoptosis and in decrease in resistance of cancer cells to chemotherapeutic agents.

Changing the membrane bilayer properties such as intrinsic curvature, elasticity and fluidity is a characteristic of several amphiphilic molecules. Statins, beyond the classical cholesterol lowering effects have been shown to alter lipid organization of artificial membranes and cell membranes in a cholesterol-independent way (Redondo-Morata et al., 2016; Penkauskas et al., 2020). This property can be included among the pleiotropic effects of statins, which are behind many benefits observed during statin therapy (Banfi et al., 2017; Oesterle et al., 2017). According to an established hypothesis, the biological properties of statins depend on their localization in the cellular membrane due to their amphiphilic properties (Mason et al., 2005; Galiullina et al., 2017). Several recent studies investigated the interactions of different statins with phospholipid membranes and their influence on the membrane structure (Sahu et al., 2019; Sariisik et al., 2019; Penkauskas et al., 2020). Statins seem to bind and influence lipid membranes, possessing different average location into the bilayer (Galiullina et al., 2019). However, a clear connection between a determined statin and the capacity to interact and alter membrane bilayer properties cannot be fully established, mainly due to the different membrane models and experimental settings used. Clinical studies in cancer patients have suggested lower cancer mortality and less side effects with lipophilic statins compared to hydrophilic ones (Ahern et al., 2011; Ahmadi et al., 2018; Beckwitt et al., 2018). In the last years the anti-tumor activity of statins was remarkably improved by using statins formulated in different drug delivery systems (Coimbra et al., 2010; Alupei et al., 2015; Safwat et al., 2017a; Matusewicz et al., 2019). In many cases the drug delivery system includes the statin in combination with a chemotherapeutic agent as doxorubicin (Pinzon-Daza et al., 2012). Indeed, the incorporation of statins in nanoparticulate drug delivery systems not only increased statins cytotoxicity but also overcame the resistance of cancer cells against common chemotherapeutic agents (Safwat et al., 2017b). This field is continuously developing, trying to identify the best carrier capable to enhance drug loading capacity, stability and therapeutic activity. According to this point of view, chitosan nanoparticles (CSNPs) are an optimal choice since they possess low toxicity and immunogenicity and good levels of biodegradability (Prabaharan, 2015). In Figure 2 are presented the different rationales behind the use of statins in cancer therapy for modulation of membrane lipids. An innovative strategy for treatment of oncological disorders is the use of amphiphilic drug-drug conjugates (ADDC), where an amphiphilic molecule, with high capacity to interact with and penetrate the lipid bilayer, is created by combining an hydrophilic anticancer drug with a hydrophobic one (Huang et al., 2014; Gao et al., 2018). Most of the times, this strategy overcomes the necessity to use a proper delivery system and since these two drugs have different pharmacokinetics, it is possible that these molecules induce synergistic pharmacological effects improving the therapeutic efficacy both *in vitro* and *in vivo*. However, ADDC is another example where the overall effect achieved in cancer therapy should not be only reconnected to the sum of the individual ones. For example, camptothecin-classical anticancer activity is related to binding to the topoisomerase-1 and DNA complex, while floxuridine, a derivative of 5-fluorouracil, is known for its high antitumor activity against cancer metastases. The combination of the hydrophobic camptothecin and hydrophilic floxuridine, used to enhance apoptosis in colon cancer cell lines creates amphiphilic molecules capable to alter the lipid bilayer properties (Hu et al., 2015). Additionally, changes in bilayer physical properties regulate membrane protein functions including the ones involved in the regulation of apoptosis (Lundbaek et al., 2010). Therefore, behind the individual molecular target of each chemotherapeutic agents, the potential effect on membrane bilayers, derived by the creation of amphiphilic molecules should be evaluated (Bruno et al., 2013; Kumar et al., 2015). A better understanding of the biological effects of chemotherapeutic drugs on lipid membranes is essential to overcome MDR since, as mentioned before, cancer cells rearrange lipid composition and organization to avoid apoptosis and resist anticancer drugs (Bernardes and Fialho, 2018; Rivel et al., 2019).

## Dietary Modification of Membrane Lipids

Several clinical studies have strongly indicated a role for fish oil and polyunsaturated fatty acids (PUFA) in cancer prevention (Caygill et al., 1996; Azrad et al., 2013). One of the main lipids present in fish oil, docosahexaenoic acid (DHA), has been shown to alter plasma membrane properties including membrane fluidity, phase behavior and permeability (Yang et al., 2011; Levental et al., 2016; Bie et al., 2020). Moreover, different studies shown that DHA can influence lipid rafts composition, altering their size or clustering capacities and consequently affecting lipid raft-regulated signaling (Shaikh et al., 2009; Turk and Chapkin, 2013; Wassall et al., 2018). Studies in mice fed with a PUFA-enriched diet shown that the molecular targets of DHA are cholesterol and sphingomyelin, two essentials building blocks of lipid rafts (Fan et al., 2003, 2004). These properties can have beneficial effects in anti-cancer therapy since behind the modification of membrane lipids, there is also a regulation of the protein receptors enriched in these membrane microdomains. For example, in breast cancer cell lines, DHA was found to influence epidermal growth factors receptors function and to enhance chemotherapy efficacy, by inducing CD95 translocation into lipid rafts, while in colon cancer cell lines it was responsible for an increase in oxidative stress and in TRAIL-induced apoptosis (Ewaschuk et al., 2012; Skender et al., 2014; Pettersen et al., 2016). These and many similar studies demonstrated two bullet-points: (1) lipid rafts play a functional role during tumorigenesis of different types of cancer (2) a therapeutic role for PUFA, since these fatty acids alter lipid raft structure/organization/function. The role for PUFA in prevention and treatment of cancer is wide and well documented, but the real efficacy of PUFA is still debated. Indeed, it is not fully established whether dietary PUFAs are integrated into raft lipids or whether their low affinity to cholesterol causes phase separation from rafts and, consequently, displacement of raft proteins (Yaqoob and Shaikh, 2010). Currently, they are mainly used in combination with different cytotoxic drugs to enhance chemotherapy efficiency (Granci et al., 2013; Newell et al., 2019). A compound reported to induce lipid rafts clustering is epigallocatechin-3-gallate (EGCG) the major polyphenol of green tea with chemo-preventives and chemo-therapeutic activities (Surh, 2003; Yang et al., 2009). However, the overall effect seems to be dependent by the tumor type since this polyphenol was observed to induce apoptosis in multiple myeloma cells (Tsukamoto et al., 2012; Huang et al., 2015), while in colon adenocarcinoma cells it increased cell viability and proliferation (Pajak et al., 2011). This discrepancy is probably related to the fact that EGCG modulates a wide spectrum of molecular targets including epidermal growth factor receptor, mitogen activated protein kinase and cyclin-dependent kinases (Khan et al., 2006; Ma et al., 2014; Fang et al., 2015). Therefore, there is not always a unique pattern of response to disruption of lipid rafts or to depletion of cholesterol from the membrane and each treatment should be evaluated in the context of the particular type of cancer and also of the specific therapeutic strategy adopted. In the last years, many scientists became interested in the evaluation of the synergistic effects of the combination of EGCG and anticancer compounds. For example, Fujiki et al. (2015) showed that the combinations of EGCG or other green tea catechins and 46 anticancer drugs synergistically induced *in vitro* anticancer effects in 58 different human cancer cell lines. Therefore, EGCG is a natural compound with proven beneficial effects both in cancer prevention and cancer therapy in combination with anticancer compounds (Fujiki and Suganuma, 2012; Fujiki et al., 2015). In Table 1 is presented the list of compounds described in this manuscript with the main mechanism of actions for MLT.

**TABLE 1:** List of compounds described in this manuscript with the related mechanism of action.

**Compound**	**Mechanism of Action**	**References**
Cyclodextrins	Cholesterol depletion	Zidovetzki and Levitan, 2007; Lopez et al., 2013; Mahammad and Parmryd, 2015
Filipin, Amphotericin, Nystatin	Cholesterol sequestration	Bittman and Fischkoff, 1972; Silva et al., 2006; Kaminski, 2014
Statins	Cholesterol synthesis inhibition/Lipid organization	Stancu and Sima, 2001; Kuipers and van den Elsen, 2007; Wang et al., 2008; Penkauskas et al., 2020
Dynasore	Cholesterol transport inhibition/Cholesterol depletion	Girard et al., 2011; Preta et al., 2015a,b
Bavituximab, PPS1D1, PC-SA	Binding to phosphatidylserine (PS)	Chalasani et al., 2015; Gerber et al., 2015; Desai et al., 2016; Desai and Udugamasooriya, 2017; De et al., 2018; Grilley-Olson et al., 2018
Duramycin, Cinnamycin	Binding to phosphatidylethanolamine (PE)	Iwamoto et al., 2007; Hullin-Matsuda et al., 2016
Cyclotides	Binding to phospholipids containing PE headgroups	Wang et al., 2012
Short chain ceramide	Increase ceramide membrane levels	Selzner et al., 2001; Stover and Kester, 2003; Stover et al., 2005; Chiantia et al., 2007
B13, LCL-464, KPB-27	Inhibition of ceramidase	Bhabak and Arenz, 2012; Bhabak et al., 2013
N,N-dimethylsphingosine	Inhibition of sphingosine kinase	Chen et al., 2014
Edelfosine, Miltefosine	Lipid rafts clustering	Gajate and Mollinedo, 2007; Gomide et al., 2013
Resveratrol, Azurin, p28 peptide	Lipid rafts organization	Delmas et al., 2013; Gao et al., 2017; Bernardes and Fialho, 2018
Amphiphilic drug-drug conjugate	Membrane lipid organization	Huang et al., 2014; Gao et al., 2018
DHA	Lipid rafts clustering/Membrane fluidity, permeability	Shaikh et al., 2009; Turk and Chapkin, 2013; Wassall et al., 2018
EGCG	Lipid rafts clustering	Tsukamoto et al., 2012; Huang et al., 2015

## Concluding Remarks and Future Directions

The better understanding of membrane lipid composition and organization gained in the last years, together with the lipidic alterations reported in tumor membranes, provides a big opportunity for cancer prevention and treatment. Nowadays, the strategy to modify membrane cholesterol/sphingolipids content is gradually replaced by a more focused approach on the modulation of membrane bilayer properties, including fluidity and elasticity, by inducing changes in the organization of lipid rafts. Rafts proteins have also an essential role in regulating lipid properties and a future field of study in MLT could be the investigation of how changes in the structural composition of raft proteins influence lipid microdomains organization. The lack of attention toward targeting these proteins as a strategy for MLT is quite surprising and it is related to the consideration of these membrane proteins as merely guests rather than as active components of lipid rafts. Further studies on these protein-lipid interactions may lead to a better understanding of the molecular mechanism of raft domains organization and may provide new strategies for their manipulation. The final aim of this modulation in cancer therapy is to increase the overall efficiency of chemotherapeutic agents, achieving a synergistic effect and defeating MDR. Studying and testing membrane-lipid targeting agents in combination with chemotherapeutic agents is a promising and innovative approach for the development of new therapeutic strategies.

## Author Contributions

The author confirms being the sole contributor of this work and has approved it for publication.

## Conflict of Interest

The author declares that the research was conducted in the absence of any commercial or financial relationships that could be construed as a potential conflict of interest.
